# Preparation, characterization, antioxidant and antianemia activities of *Poria cocos* polysaccharide iron (III) complex

**DOI:** 10.1016/j.heliyon.2023.e12819

**Published:** 2023-01-07

**Authors:** Yue Zhang, Jiajing Huang, Mingjie Sun, Yuting Duan, Lei Wang, Nianjun Yu, Daiyin Peng, Weidong Chen, Yanyan Wang

**Affiliations:** a School of Pharmacy, Anhui University of Chinese Medicine, Hefei, China; b MOE-Anhui Joint Collaborative Innovation Center for Quality Improvement of Anhui Genuine Chinese Medicinal Materials, Hefei, China; c Institute of Conservation and Development of Traditional Chinese Medicine Resources, Hefei, China; d Anhui Province Key Laboratory of Chinese Medicinal Formula, Hefei, China

**Keywords:** *Poria cocos* polysaccharide, Polysaccharide-iron (III) complex, Iron supplement, Characterization, Antioxidant activity

## Abstract

As a new natural antioxidant with high safety and non-toxic side effects, polysaccharide can also be used as a critical macromolecular carrier to form a stable iron complex with Fe^3+^. Our previous study has extracted and purified the homogeneous polysaccharide (PCP1C) from *Poria cocos*. In this study, the PCP1C-iron (III) complex was synthesized by co-thermal synthesis with PCP1C and ferric trichloride. The chelating capacity, iron releasing capacity, and qualitative identification of complex were evaluated. The complex was characterized by scanning electron microscope-energy dispersive spectrometer (SEM-EDS) analysis, particle size distribution, and fourier transform infrared (FTIR) spectroscopy. The antioxidant and iron supplement effects of the complex were also studied *in vitro* and in the iron deficiency anemia (IDA) rat model. The results showed that the iron content in the PCP1C-iron (III) complex was 28.14% with no free iron, and the iron release rate was 95.3%. The structure analysis showed that the iron core of the PCP1C-iron (III) complex existed in the form of β-FeOOH and the surface of the complex become smooth and particle size increased, which indicated the high iron content of polysaccharide iron and slow release. Furthermore, we found that the PCP1C iron (III) complex had positive scavenging effect on DPPH, ABTS, MDA, and hydroxyl radical *in vitro* study and significantly increased the levels of red blood cell (RBC), Hemoglobin (Hb), and red blood cell specific volume (HCT) in IDA rat model. Therefore, our results suggested that the PCP1C-iron (III) complex is expected to develop into a new comprehensive iron supplement and antioxidant.

## 1 Introduction

Iron is one of the indispensable trace elements in the human body which participates in the composition, activation, and metabolism of various enzymes and maintains the normal operation of various physiological activities [1]. Iron deficiency anemia (IDA) is a nutritional deficiency disorder mainly related to the patient's long-term lack of iron intake, rapid iron loss, or abnormal gastrointestinal absorption function [2]. In severe cases, IDA can lead to immunosuppression, pallor, dizziness, fatigue, and other side effects. For IDA patients, iron supplements are commonly performed.

The first generation of iron supplements are mainly inorganic iron compounds, of which Fe_2_SO_4_ is the most commonly used. Although iron content is high in the first generation of iron supplements, there are severe gastrointestinal irritation and other adverse effects [3]. The second generation of iron supplements are mainly soluble small molecule organic iron salts represented by ferrous lactate, ferrous gluconate, etc. Although the adverse reactions were reduced, problems such as iron instability also exist [3]. Therefore, many researchers are committed to developing the third generation of macromolecular iron complexes, such as polypeptide iron chelate, heme iron, and polysaccharide iron, etc. Among them, polysaccharide iron represented by Niferex has attracted considerable attention because of its stable structure, high bioavailability, and high iron content [4]. In addition, polysaccharide iron not only has the activity of supplementing iron, but also has other beneficial activities of polysaccharide [5–8]. Although polysaccharide iron has been recognized as a potential drug to treat IDA, the clinical application of polysaccharide iron prepared from plant polysaccharides is limited at present, which may be related to the unclear activity of polysaccharide iron and the low purity of polysaccharide.

*Poria cocos* (Schw.) Wolf is a traditional Chinese medicine and has been widely used in Asia for thousands of years. In the 2020 edition of the Chinese Pharmacopoeia, there are 1587 kinds of prescription preparations and single preparation, 260 of which contain *Poria cocos*, accounting for 16%. In addition, *Poria cocos* is also widely used as food [9,10,11,12]. Modern pharmacological research shows that the polysaccharides isolated from *Poria cocos* have many biological activities, including anti-tumor [13], anti-inflammatory [14], liver protection [9], and immunity enhancement [15,16], especially antioxidant effects [17]. A new *Poria cocos* polysaccharide named PCP1C was purified from *Poria cocos* sclerotia in our previous study which was composed of galactose, glucose, mannose, and fucose [18]. The dried PCP-1C contained carbohydrates of 97.54 ± 1.64%. Mw (kDa) = 19.2, Mn (kDa) = 17. The value of Mw/Mn was close to 1, which indicates that PCP-1C was relatively homogeneous. Further experiments showed that PCP1C had excellent anti-inflammatory and antioxidant activity [19]. However, whether it is possible to use PCP1C with higher purity to prepare polysaccharide iron and how its physiological activity needs to be further studied.

In this study, single factor investigation and orthogonal experiment were used to optimize the synthesis process of PCP1C-iron (III) complex. Qualitative evaluation was performed from the aspects of general physical and chemical properties, qualitative identification and *in vitro* release capacity. Its structure was characterized by SEM-EDS analysis, particle size distribution, and FTIR spectroscopy. Besides, the antioxidation effects and *anti*-IDA effects of the PCP1C-iron (III) complex were studied. The results showed that the PCP1C-iron (III) complex contained no free iron, the iron content in the complex was 28.14%, and the iron release rate was 95.3%. The structure analysis showed that PCP1C could form complexes with Fe^3+^ through hydroxyl and carbonyl groups. The surface of PCP1C iron (III) complex became smooth and the particle size increased. The antioxidant study showed that the PCP1C iron (III) complex had an excellent scavenging effect on DPPH, ABTS, MDA, and hydroxyl radicals. In addition, the PCP1C iron (III) complex had a positive therapeutic effect on IDA rats, significantly increasing the levels of RBC, Hb and HCT. Taken together, our research showed that the PCP1C iron (III) complex is expected to develop into a new comprehensive iron supplement and antioxidant.

## 2 Materials and methods

### 2.1 Materials and reagents

The *Poria cocos* sclerotia was collected from Jinzhai County, LuAn City, Anhui Province, China. Ferric chloride (FeCl_3_), trisodium citrate dihydrate (C_6_H_5_Na_3_O_7_), ammonium iron (II) sulfate [Fe(NH_4_)_2_·(SO_4_)_2_·6H_2_O], potassium hexacyanoferrate (II) (K_4_ [Fe(CN)_6_]), potassium thiocyanate (KSCN) and *o*-phenanthroline (C₁₂H₈N₂) were purchased from Tianjin Guangfu Technology Development Co., Ltd. Pepsin, trypsin, 2,2-diphenyl-1-picrylhydrazyl (DPPH), 2-ethylbenzothiazoline sulphonic acid-6 ammonium salt (ABTS) were purchased from Shanghai Yuanye Biotechnology Co., Ltd. Niferex capsule (150 mg/capsule) was manufactured by Kermers Urban Pharmaceuticals Inc, USA., Malondialdehyde (MDA) test kit was obtained from Shanghai Yaji Biotechnology Co., Ltd. AOAC low-iron diets (iron content≤ 2.3 mg kg^−1^) were gained from Beijing Botai Hongda Biotechnology Co., Ltd. The water used in the experiment was ultrapure water, and the reagents were analytical alcohols.

### 2.2 Preparation and purification of PCP1C

PCP1C was isolated and purified by our previous experimental method [18]. Briefly, *Poria cocos* sclerotium powder was extracted at 65 °C and concentrated to obtain water extract. 95% ethanol was used for preliminary purification, and the precipitate was centrifuged. The precipitated polysaccharide was deproteinized by the sevag method, and then the dialysis bag (molecular weight 3500 da) was further used to remove small molecular impurities. Finally, the PCP1C was purified by a Cellulose DEAE-52 column and further eluted with ultrapure water on the Sephacryl S-500 column.

### 2.3 Synthesis method

Based on the previous method [8], the influence of reaction time, dosage of trisodium citrate, solvent volume, pH value, reaction temperature, and dosage of ferric chloride to PCP1C iron (III) complex was systematically studied by the single factor method. The optimal synthesis process of the PCP1C iron (III) complex was further determined by an orthogonal experiment. The final synthetic process was as follows: PCP1C (1.0 g), trisodium citrate (0.5 g) and 1 mL of 10% sodium carbonate were added in 40 mL aqueous solution at 70 °C. 2 moL·L^−1^ FeCl_3_ solution and 6 moL·L^−1^ NaOH solution were dropped into the reaction system and fully stirred. The pH of the reaction solution was controlled at 9. After 1 h of reaction, the cooled reaction solution was centrifuged at 3000 r·min^−1^ for 15 min, and the upper layer of reddish-brown liquid was collected. Anhydrous ethanol was added to the liquid with a ratio of 1:1, shaken, and kept at 4 °C overnight. After centrifugation at 3000 r·min^−1^ for 15 min, the precipitate was collected, washed with an appropriate amount of absolute ethanol, and centrifuged again. Finally, the precipitate was centrifuged at 3000 r·min^−1^ for 15 min and freeze dried.

### 2.4 Physical evaluation of PCP1C-iron (III) complex

#### 2.4.1 Iron (III) chelating capacity

Based on the literature method [20], iron content was determined by *o*-phenanthroline Spectrophotometry. Accurately suck 0.2, 0.4, 0.6, 0.8, 1.0, and 1.2 mL of Fe (II) standard solution into 50 mL volumetric flasks, respectively, and add 1.5 mL of 8% ascorbic acid solution and 1.5 mL of 0.1% *o*-phenanthroline color developing solution respectively. Ultrapure water was used to fix the volume to 50 mL. Take the reagent solution as blank, use the ultraviolet visible spectrophotometer to measure the absorbance value at 510 nm, and fit the regression equation. PCP1C iron (III) complex solution (200 μg mL^−1^) was prepared accurately. 5 mL of PCP1C iron (III) complex solution were placed in a 25 mL volumetric flask. Ascorbic acid solution and phenanthroline color developing solution was added to the reaction system according to the same method. Finally, the iron content is calculated according to the fitted linear regression equation.

#### 2.4.2 Qualitative identification

In order to identify whether there is free Fe^3+^ in the PCP1C-iron (III) complex, the synthesized PCP1C-iron (III) complex and Fe(OH)_3_ were qualitatively compared in aqueous solution, NaOH solution, KSCN solution, and K_4_ [Fe(CN)_6_] solution [21].

#### 2.4.3 Iron release properties

1.0 g PCP1C-iron (III) complex was placed in 1000 mL simulated gastric fluid for 120 min at 37 °C. The sample was detected by the 1,10-phenanthroline colorimetric method every 30 min at 510 nm. After simulated gastric fluid [22] degradation for 120 min, it was evaporated under a nitrogen flow at 37 °C. Then, 1000 mL simulated intestinal fluid was added. The sample was passed through a 0.45 μm filter membrane and tested by the 1,10-phenanthroline colorimetric method within 3 h. The iron content was determined by a linear regression equation. The iron release rate is calculated as follows: Iron release rate (%) = $\frac{A1}{A0}\times 100\%$, where A_0_ was the iron content of PCP1C-iron (III) complex, and A_1_ was the total amount of iron in the solution.

### 2.5 Structural characteristics

#### 2.5.1 SEM-EDS analysis

Take appropriate amounts of dried PCP1C and PCP1C-iron (III) complex, stick them to the carbon conductive dielectric plate and gild them (K5758-264, Emitech High Vacuum Ion Sputterer), and then use the scanning electron microscope to scan at room temperature to observe the microscopic morphology of the sample (Quanta FEG 250, Frequency Electronics, Inc.). The mass percentages of surface elements of PCP1C and PCP1C-iron (III) complex were analyzed by energy dispersive spectrometer (INCA X-MAX50, Oxford Instruments).

#### 2.5.2 Particle size distribution

Take appropriate PCP1C and PCP1C-iron (III) complex powder and dilute them to 0.1 mg L^−1^ with ultrapure water, then the particle size distributions were measured by a nano-particle size analyzer (ZEN3690, USA).

#### 2.5.3 FTIR analysis

PCP1C-iron (III) complex (2 mg) and PCP1C (2 mg) were mixed with 200 mg KBr *respectively, then tableted, and scanned at 4000 ∼* 500 cm^−1^ by *Infrared spectrometer* (Nicolet nexus 410, Shanghai Rongrui Scientific Instrument Co., Ltd).

### 2.6 Experiment on antioxidation

#### 2.6.1 DPPH radical scavenging activity

Slightly modified based on literature [23], a series of aqueous solutions of PCP1C, PCP1C iron (III) complexes and VC with different concentrations were prepared by ultrapure water. 1.5 mL solution with 1.5 mL DPPH (25 mg L^−1^) ethanol solution was mixed and incubated at 25 °C for 30 min. The absorbance of the solution was measured at 517 nm. The calculation method was shown in formula (1). In the formula: A_0_ represents the absorptance of the DPPH diluent. A_1_ represents absorptance of solutions after mixed reaction. A_2_ represents the absorptance of the original sample solution.

(1) $$\eta \;\left(\%\right)=\left(1-\frac{A1-A2}{A0}\right)*100\%$$

#### 2.6.2 Hydroxyl radical scavenging activity

According to the reported method [6], 1.0 mL of PCP1C, PCP1C iron (III) complex and VC aqueous solution with different concentrations were mixed with 1.0 mL of FeSO_4_ (9 mmol L^−1^), respectively. 1.0 mL of salicylic acid ethanol solution (9 mmol L^−1^) and 0.05 mL of H_2_O_2_ (9 mmol L^−1^) were added to the reaction system and reacted for 30 min at 37 °C. Finally, the absorbance of the solution was measured at 510 nm. The calculation method was shown in formula (1). A_0_ represents the absorptance of the mixed solution except sample solution. A_1_ represents the absorptance of the solutions after mixed reaction. A_2_ represents the absorptance of the original sample solution.

#### 2.6.3 ABTS free radical scavenging activity

Based on the reported method [7], PCP1C-iron (III) complex, PCP1C, and VC solution were configured into a series of different sample solutions by using ultrapure water. ABTS free radical stock solution was prepared by adding 440 μL of K_2_S_2_O_8_ solution (140 mmol L^−1^) to 25 mL of ABTS radical solution (7 mmol L^−1^) and protected from light for 12 h. The 2.7 mL of ABTS free radical dilution and 0.3 mL of sample solutions were mixed. After 10 min of reaction, the absorbance of the solution was measured at 734 nm. The calculation method was shown in formula (1). In the formula: A_0_ represents the absorptance of the ABTS diluent. A_1_ represents the absorptance of the solutions after mixed reaction. A_2_ represents the absorptance of the original sample solution.

#### 2.6.4 Inhibition of malondialdehyde (MDA) activity

Slightly modified based on literature [24], liver tissue was taken from mice after ether anesthesia. Then 4% (w/v) liver homogenate was prepared. 1 mL sample solution or VC solution with different concentrations to the mouse liver homogenate was added and incubated in 37 °C water for 1.5 h. Then, 1 mL of trichloroacetic acid (10%, w/v) and 1 mL of thiobarbituric acid (0.67%, w/v) were added to the mixture. The supernatant was centrifuged after 15 min of incubation, and then ultraviolet spectrophotometer was used to measure the absorbance of the supernatant at 532 nm. The calculation method was shown in formula (1). A_0_ represents the absorptance of the mixed solution except sample solution. A_1_ represents the absorptance of the solutions after mixed reaction. A_2_ represents the absorptance of the original sample solution.

### 2.7 Treatment of IDA with PCP1C-iron (III) complex

Young male Wistar rats (4-week-old, SPF) were purchased from the experimental animal center of Anhui Medical University (SCXK-2020-001). After 7 days of adaptive feeding, standard feed and drinking water were given. Rats were maintained at the room temperature (24 ± 2 °C), the relative humidity 55 ± 5%, and 12 h dark-light cycle. All animal experimental procedures were approved by the Experimental animal ethics committee of Anhui University of Chinese medicine (Approval number: AHUCM-mouse-2021,033). All animal care and experimental procedures were carried out conformed to the Guide for the Care and Use of Laboratory Animals.

Forty-eight Wistar rats were assigned to the normal group (twelve rats) and model group (thirty-six rats) randomly. The rats in the model group received ocular bloodletting three times a week and were fed an AOAC low iron diet. The whole molding cycle lasted for one month. The rats in the normal group had no special intervention and drank and ate normally. IDA was defined as Hb < 100 g L^−1^ [25]. After one month, six rats in each group were randomly selected for blood routine tests for the model validation. Subsequently, the rats with successful modeling were randomly divided into model group, positive control group (Niferex with 30 mg kg^−1^), low, middle, and high dose group with PCP1C-iron (III) complex (15, 30, 60 mg kg^−1^). The treatment period was one month. During the treatment, all groups were fed with AOAC low iron diet except the normal group. Fasting for 12 h after the last intragastric administration, all rats were anesthetized with 20% Ethyl carbamate (5 mL kg^−1^), and blood was taken from the abdominal aorta for a routine blood test by an automated hematology analyzer (MEK-8222 K Nihon Kohden, Japan).

### 2.8 Statistics analysis

The data were expressed by mean ± SD and statistically analyzed by SPSS 23.0 software. T-test is used between two groups of samples, one-way ANOVA is used for more than two groups, and LSD test is used for pairwise comparison between multiple groups. *P* < 0.05 means the difference is significant.

## 3 Results and discussion

### 3.1 Iron (III) chelating capacity

Previous studies have shown that polysaccharides have different iron complexing capacities due to their different structures and molecular weights [26,27]. In the preparation of the PCP1C-iron (III) complex, the optimum preparation process of polysaccharide iron was determined by a single factor investigation (Supplementary Figure 1) and an orthogonal experiment (Supplementary Table 1). According to the equation of the calibration curve (Y = 121.39X+0.1164) with a correlation coefficient of 0.9992, the iron content of the PCP1C-iron (III) complex was 28.14%.

### 3.2 Qualitative identification

**Fig. 1:**
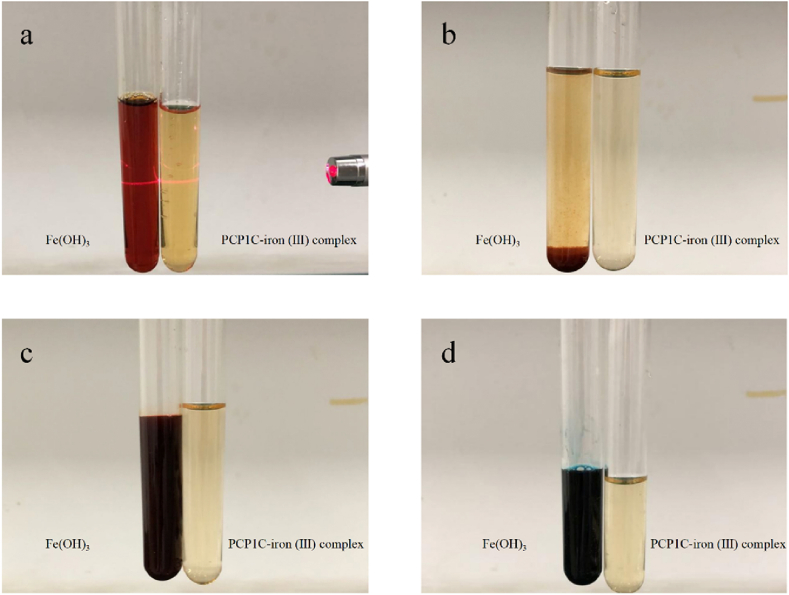
Comparison of Fe (OH)_3_ and PCP1C-iron (III) complex in physicochemical properties. (a) Aqueous solution, (b) NaOH solution, (c) KSCN solution, (d) K_4_ [Fe (CN)_6_] solution.

The PCP1C-iron (III) complex obtained under the optimum conditions was an amorphous reddish brown powder that was odorless, freely soluble in water and neutral in aqueous solution (Fig. 1a). When NaOH, K_4_ [Fe (CN)_6_], and KSCN were added to the aqueous solution of the PCP1C-iron (III) complex or Fe (OH)_3_, respectively, the precipitation (Fig. 1b), dark blue precipitate (Fig. 1c) and blood-red floccules (Fig. 1d) appeared in Fe (OH)_3_ aqueous solution, but not in the PCP1C-iron (III) complex. These results clearly showed that the PCP1C iron (III) complex exists in the form of a complex rather than free iron ions [20].

### 3.3 Release properties *in vitro*

When administered orally, the release of the drug directly affects its efficacy [28]. After oral administration, bound iron is released in the gastric and intestinal environment and absorbed in the small intestine [29]. Studies have shown that polysaccharide iron has a good iron release efficiency in the stomach and intestine [30].

**Fig. 2:**
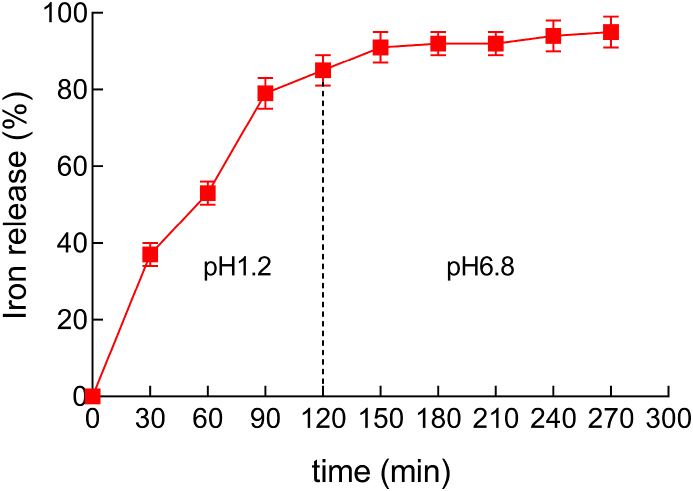
Iron release from the PCP1C-iron (III) complex in simulated gastric juice and simulated intestinal fluid medium (mean ± SD, n = 3).

In this study, the iron release of PCP1C-iron (III) complex was verified in simulated gastric juice (pH 1.2) and simulated intestinal fluid medium (pH 6.8) [31]. As shown in Fig. 2, in the first 120 min, almost 85% of the complex iron was released into the release medium, indicating that the polysaccharide iron was mainly slowly released in the stomach tissue. After 5 h of digestion under simulated intestinal conditions, the iron release rate of polysaccharide iron reached 95.3%. These results indicated that the PCP1C-iron (III) complex exhibited good water solubility in gastrointestinal environment, which was conducive to the absorption of iron by the human body and had good bioavailability.

### 3.4 Characterization of PCP1C-iron (III) complex

#### 3.4.1 SEM-EDS analysis

**Fig. 3:**
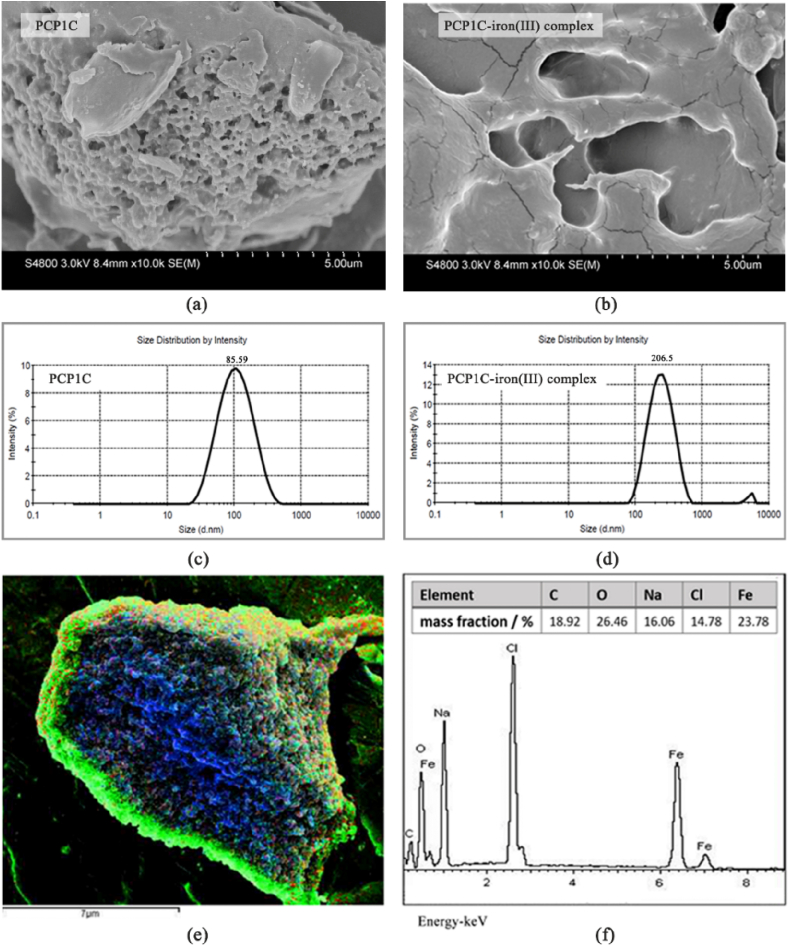
Characterization of PCP1C-iron (III) complex. The SEM images of PCP1C (a) and PCP1C-iron (III) complex (b), the particle size distribution of PCP1C (c) and PCP1C-iron (III) complex (d), the EDS images of PCP1C-iron (III) complex, yellow represents element Fe (e), Analysis of PCP1C-iron (III) complex energy spectrum (f).

The surface morphology of the PCP1C and PCP1C-iron (III) complex was measured using SEM-EDS analysis. As shown in Fig. 3a, the results showed that PCP1C had a loose network structure similar to honeycomb, with different particle sizes. However, when PCP1C was complexed with Fe^3+^, its surface become flat and smooth with a smaller (Fig. 3b). The aggregation of polysaccharides may be due to the complexing force between polysaccharides and Fe^3+^. As shown in Fig. 3e, the elemental distribution map indicated that iron was widely distributed in the PCP1C-iron (III) complex. The energy spectrum analysis showed that the PCP1C-iron (III) complex was rich in iron, and the mass fraction was 23.78% (Fig. 3f), which further indicated that PCP1C formed a complex with iron.

#### 3.4.2 Particle size distribution

The particle size distributions of PCP1C and PCP1C-iron (III) complex were shown in Fig. 3c and d, and the average particle sizes of PCP1C and PCP1C-iron (III) complex were mainly distributed at 85.59 nm and 206.5 nm, respectively. Combined with the SEM-EDS analysis, the results show that compared with the PCP1C, the particle size of the PCP1C-iron (III) complex was significantly increased, and it was inferred that the hydroxyl group on the polysaccharide chain was complexed with the iron, so that the polysaccharide chain was opened and recombined around the formed iron core, resulting in an increase in the particle size of the complex.

#### 3.4.3 FTIR analysis

**Fig. 4:**
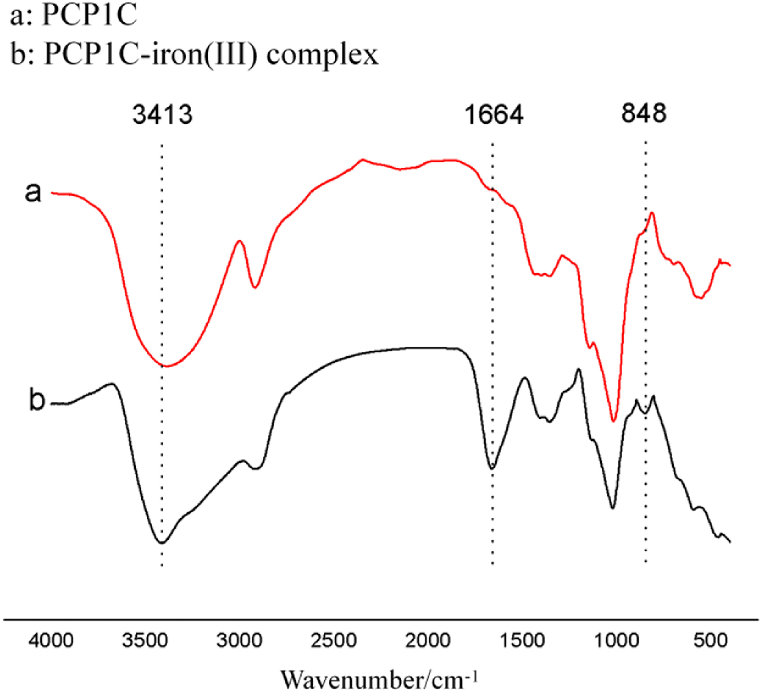
Infrared spectrogram of PCP1C and PCP1C-iron (III) complex. The red line represents PCP1C (a), and the black line represents PCP1C-iron (III) complex (b).

The infrared spectrogram of PCP1C and PCP1C-iron (III) complex were shown in Fig. 4. The absorption peak of the PCP1C-iron (III) complex at 850 cm^−1^ indicated that the iron in PCP1C-iron (III) complex existed in the form of the β-FeOOH iron core [22]. The carbonyl absorption peak at 1666 cm^−1^ indicated that Fe (III) complexes with carbonyl caused a change in carbonyl vibration dipole moment [5]. The absorption peak at 3413 cm^−1^ was the vibration absorption peak of the hydroxyl group in the polysaccharide molecule [32], and the absorption peak was narrowed after complexing with Fe (III), indicating that the hydroxyl group in the polysaccharide participated in the complexation reaction.

The above results showed that PCP1C could react with Fe^3+^, and the iron core of polysaccharide iron existed in the form of β-FeOOH. Due to the presence of Fe^3+^, the surface of polysaccharide iron became smooth and the particle size increased, which may be closely related to the slow release of PCP1C-iron (III) complex.

### 3.5 Antioxidant activities of the PCP1C-iron (III) complex

#### 3.5.1 DPPH radical scavenging activity

**Fig. 5:**
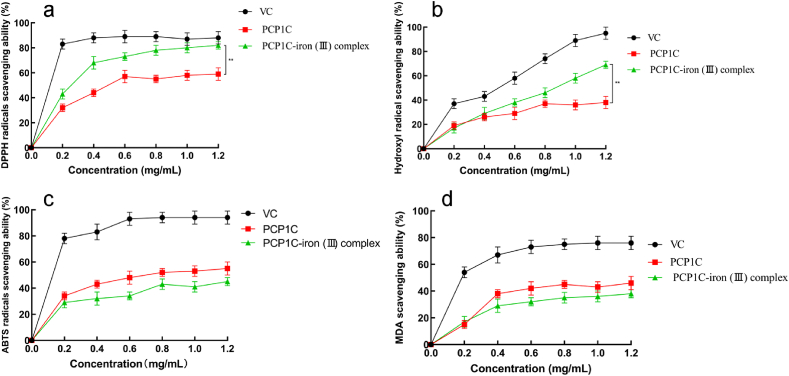
The antioxidant capacity of PCP1C-iron (III) complex and PCP1C (mean ± SD, n = 6). (a) DPPH radicals scavenging activity, (b) Hydroxyl radicals scavenging activity, (c) ABTS radicals scavenging activity, (d) MDA scavenging activity. **p < 0.01 represents significance between the two groups.

A variety of human diseases are associated with free radicals, including inflammation, cancer, diabetes, and more. Free radicals are strong oxidants and once accumulated in the human body, which will accelerate cell senescence and even apoptosis [33]. The scavenging effect of DPPH radical is widely used to quantitatively determine the *in vitro* antioxidant capacity of biological samples, pure compounds and extracts. In previous studies, the polysaccharide iron complex has strong DPPH radical scavenging activity [7]. As shown in Fig. 5a, the current results showed that both PCP1C and PCP1C-iron (III) complex had DPPH radical scavenging activity in a concentration-dependent manner within a certain concentration range (0–1.2 mg mL^−1^). Importantly, PCP1C-iron (III) complex had stronger DPPH radical scavenging activity compared to the PCP1C. When the concentration was 1.2 mg mL^−1^, the scavenging rates of DPPH radicals by VC, PCP1C, and PCP1C-iron (III) complex were 88.40%, 59.62%, and 83.73%.

#### 3.5.2 Hydroxyl radical scavenging activity

Hydroxyl radical (-OH) is a kind of free radical with extremely strong oxidation ability and has the ability to destroy biological cells, leading to cell death or tissue damage, especially cell membrane damage [34]. When the concentration was 1.2 mg mL^−1^, the scavenging rates of VC, PCP1C, and PCP1C-iron (III) complex were 95.40%, 38.62%, and 69.73%, respectively (Fig. 5b). The order of scavenging hydroxyl radicals was: VC > PCP1C-iron (III) complex > PCP1C, which indicated that the PCP1C-iron (III) complex had stronger hydroxyl radical scavenging ability than PCP1C.

#### 3.5.3 ABTS radical activity

ABTS is a free radical centered on the nitrogen atom, which is very stable *in vitro* and is often used to detect the antioxidant activity of substances *in vitro* [35]. As shown in Fig. 5c, when the concentration was in the range of 0 mg mL^−1^ to 1.2 mg mL^−1^, the scavenging effects of PCP1C-iron (III) complex, PCP1C, and VC on ABTS free radicals increased with increasing concentration. When the concentration was 1.2 mg mL^−1^, the scavenging rates of VC, PCP1C, and PCP1C-iron (III) complex on ABTS radicals were 94.40%, 79.5%, and 69.43%, respectively. These results indicated that PCP1C and PCP1C-iron (III) complexes had significant scavenging ability for ABTS free radicals.

#### 3.5.4 MDA activity in mouse liver

Oxygen free radicals are products of aerobic metabolism, and MDA, the end product produced by the lipid peroxidation reaction initiated by them, can cause a series of pathological damage to the body by affecting protein denaturation and genetic information mutation of cells [36]. The degree of lipid peroxidation can be embodied by measuring MDA, thus indirectly reflecting the level of oxidative stress. In our study, we found that the inhibitory ability was VC > PCP1C > PCP1C-iron (III) complex (Fig. 5d). In general, the PCP1C-iron (III) complex still showed good free radical scavenging ability to inhibit MDA production to a certain extent.

In short, we evaluated the antioxidant activity of PCP1C and PCP1C-iron (III) complex by measuring DPPH scavenging activity, hydroxyl radical activity, ABTS activity, and MDA activity. The above results showed that the PCP1C-iron (III) complex could still retain the antioxidant effect of PCP1C and may even present a stronger antioxidant effect than PCP1C.

### 3.6 Iron supplement effect of PCP1C-iron (III)

#### 3.6.1 Establishment of IDA model

Research showed that plant polysaccharides can form a stable complex with iron, and the concentration of iron is relatively high under physiological conditions with less toxic and side effects, so it is an ideal iron supplement for the treatment of IDA [6,7,37]. This study investigated the therapeutic effect of PCP1C-iron (III) complex on iron deficiency anemia in rat model.

IDA refers to the reduction of systemic iron reserves that may occur in the case of increased demand, reduced intake, reduced or poor absorption, or chronic blood loss [38]. A low iron diet with periodic orbital blood collection is one of the common methods to establish animal models of IDA [25] and was therefore the model of choice for the current study. Compared to the normal group, we found that the rats in the model group showed sluggish responses, slow growth, rough and sparse hair. As shown in Table 1, hematology indicators results showed that RBC, Hb and HCT were significantly decreased in the model group. According to the literature [25], when Hb < 100 g L^−1^, the indicators of the iron deficiency anemia rat model were statistically significant, indicating that the IDA model was successfully established.

**Table 1:** Changes of various indexes of rat after modeling (mean ± SD, n = 6).

Groups	Hb (g·L^−1^)	RBC (10^12^ L^−1^)	HCT (%)	Body weight (g)
Normal	173.27 ± 3.21	10.72 ± 0.07	55.17 ± 2.55	197.12 ± 10.34
Model	83.33 ± 4.41##	5.76 ± 0.61##	25.53 ± 2.23##	150.65 ± 8.76##

Note: ^##^*P* < 0.01 compared to normal group.

#### 3.6.2 Anti IDA effect of PCP1C iron (III) complex

The detection of RBC, Hb, and HCT are helpful for diagnosing various types of anemia, guiding the treatment method, and monitoring the treatment effect of anemia [27]. Compared to the normal group, the RBC, Hb, and HCT levels of rats in the model group decreased significantly. After treatment with the PCP1C-iron (III) complex, these indexes were increased in a dose-dependently manner (Table 2). In addition, the body weight of rats in the PCP1C iron (III) complex group was higher than that in the model group (*P* < 0.01), possibly because the lack of iron affected the activity of enzymes or the oxygen supply to cells, thereby reducing the energy metabolism of the body. What's more, the PCP1C-iron (III) complex (60 mg kg^−1^) showed more efficiency than the Niferex group, which may be related to the good iron release performance of the PCP1C-iron (III) complex. In conclusion, our results showed that the PCP1C-iron (III) complex had significant therapeutic effect on IDA.

**Table 2:** Changes in blood routine index and body weight (mean ± SD, n = 6).

Groups	RBC (g·L^−1^)	Hb (g·L^−1^)	HCT (%)	Body weight (g)
Normal	9.43 ± 0.17	166.50 ± 4.92	48.22 ± 1.44	269.23 ± 12.56
Model	6.99 ± 0.32##	97.20 ± 4.22##	30.73 ± 1.13##	183.27 ± 16.64##
Niferex (30 mg kg ^−1^)	8.91 ± 0.37**	163.20 ± 3.61**	43.50 ± 1.28**	243.96 ± 18.55**
PCP1C-iron (III) complex (15 mg kg ^−1^)	8.12 ± 0.27*	158.80 ± 1.82**	40.57 ± 1.14**	219.58 ± 16.26**
PCP1C-iron (III) complex (30 mg kg ^−1^)	9.23 ± 0.29*	159.80 ± 4.01**	47.23 ± 0.91**	231.00 ± 17.93**
PCP1C-iron (III) complex (60 mg kg ^−1^)	10.41 ± 0.51*	176.20 ± 4.01**	49.53 ± 1.58**	259.88 ± 14.28**

Note: ^#^*P* < 0.05, ^##^*P* < 0.01 compared to normal group. *P < 0.05, **P < 0.01 compared to model group.

## 4 Conclusions

**Fig. 6:**
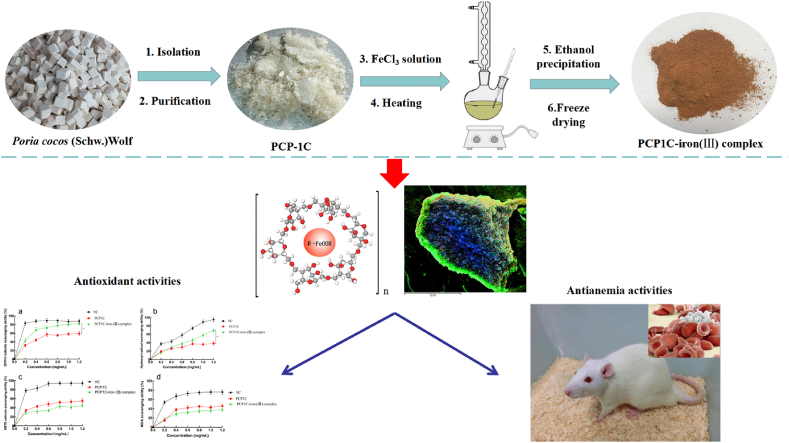
Preparation, characterization, antioxidant and antianemia activities of the PCP1C-iron (III) complex.

In this study, we used PCP1C with high purity, high homogeneity, and obvious pharmacodynamic activity to synthesize the PCP1C-iron (III) complex, which had less impurities and is more likely to be clinically transformed. The iron core of the PCP1C-iron (III) complex existed in the form of β-FeOOH. PCP1C-iron (III) complex had the positive iron chelating ability and iron releasing ability. Besides, the PCP1C-iron (III) complex also displayed antioxidant properties and iron supplementation effect. These findings indicate that the PCP1C-iron (III) complex is expected to become a new multifunctional iron supplement and antioxidant. The graphic summary was shown in Fig. 6. In addition, the structure of polysaccharide iron complex is complex, and more advanced technologies and research methods will be used later to obtain a clearer molecular structure. At the same time, its toxicity and clinical application also need further study.

## Author contributions

Yue Zhang: performed the experiments and wrote the paper; Jiajing Huang and Mingjie Sun: performed the experiments; Yuting Duan and Lei Wang: analyzed and interpreted the data; Nianjun Yu and Daiying Peng: contributed reagents, materials, analysis tools or data; Yanyan Wang and Weidong Chen: conceived and designed the experiments.

## Declaration of competing interest

The authors declare that they have no conflict of interest.
