# Supplementary material for: Preparation, characterization, antioxidant and antianemia activities of Poria cocos polysaccharide iron (III) complex
Source: Heliyon. 2023 Jan 7;9(1):e12819. doi: 10.1016/j.heliyon.2023.e12819 (PMC9840143; doi:10.1016/j.heliyon.2023.e12819)
Supplement: Multimedia component 1 [file mmc1.docx]

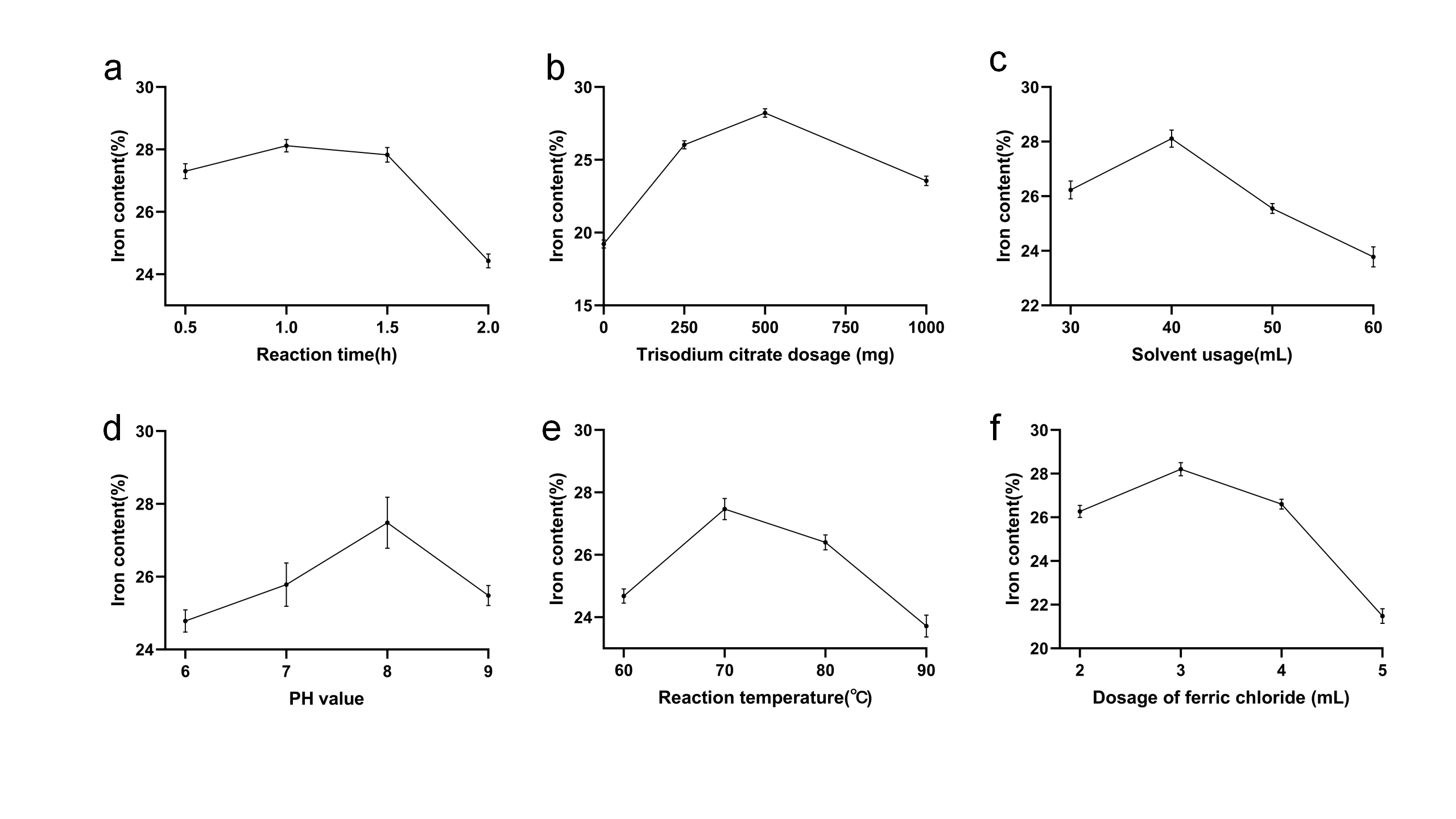


Supplementary Figure 1 Effects on the iron content of PCP1C-iron (III) complex: (a) reaction time, (b) teisodium citrate dosage, (c) solvent volume, (d) pH value, (e) reaction temperature, (f) dosage of ferric chloride. Each value is the mean ± SD of triplicate measurements (n = 3).

Supplementary table 1 Orthogonal array design L9(3^4^) and the iron content of PCP1C-iron (III) complex.

| level | Temperature/℃  A | Solid-liquid ratio  B | pH  C | Reaction time /min  D | | |
| --- | --- | --- | --- | --- | --- | --- |
| 1 | 65 | 1:35 | 7 | 40 | |  |
| 2 | 70 | 1:40 | 8 | 60 | |  |
| 3 | 75 | 1:45 | 9 | 80 | |  |
| test | A | B | C | D | | iron content/% |
| 1 | 1 | 1 | 1 | 1 | | 16.25±0.21 |
| 2 | 1 | 2 | 2 | 2 | | 23.57±0.18 |
| 3 | 1 | 3 | 3 | 3 | | 22.87±0.16 |
| 4 | 2 | 1 | 2 | 3 | | 25.11±0.25 |
| 5 | 2 | 2 | 3 | 1 | | 28.08±0.13 |
| 6 | 2 | 3 | 1 | 2 | | 19.19±0.16 |
| 7 | 3 | 1 | 3 | 2 | | 27.69±0.21 |
| 8 | 3 | 2 | 1 | 3 | | 18.24±0.14 |
| 9 | 3 | 3 | 2 | 1 | | 20.28±0.21 |
| K_1_ | 20.897 | 23.017 | 17.893 | 21.537 |  |  |
| K_2_ | 24.127 | 23.297 | 22.987 | 23.483 |  |  |
| K_3_ | 22.070 | 20.780 | 26.213 | 22.073 |  |  |
| R | 3.230 | 2.517 | 8.320 | 1.946 |  |  |
| optimum | A2 | B2 | C3 | D2 |  |  |
